# Sinomenine Hydrochloride Ameliorates Fish Foodborne Enteritis *via* α7nAchR-Mediated Anti-Inflammatory Effect Whilst Altering Microbiota Composition

**DOI:** 10.3389/fimmu.2021.766845

**Published:** 2021-11-23

**Authors:** Jiayuan Xie, Ming Li, Weidong Ye, Junwei Shan, Xuyang Zhao, You Duan, Yuhang Liu, Bruno Hamish Unger, Yingyin Cheng, Wanting Zhang, Nan Wu, Xiao-Qin Xia

**Affiliations:** ^1^ Institute of Hydrobiology, Chinese Academy of Sciences, Wuhan, China; ^2^ College of Advanced Agricultural Sciences, University of Chinese Academy of Sciences, Beijing, China; ^3^ College of Fisheries and Life Science, Dalian Ocean University, Dalian, China

**Keywords:** SBMIE, zebrafish, sinomenine hydrochloride, α7nAChR, anti-inflammation, microbiota

## Abstract

Foodborne intestinal inflammation is a major health and welfare issue in aquaculture. To prevent enteritis, various additives have been incorporated into the fish diet. Considering anti-inflammatory immune regulation, an effective natural compound could potentially treat or prevent intestinal inflammation. Our previous study has revealed galantamine’s effect on soybean induced enteritis (SBMIE) and has highlighted the possible role of the cholinergic anti-inflammatory pathway in the fish gut. To further activate the intestinal cholinergic related anti-inflammatory function, α7nAchR signaling was considered. In this study, sinomenine, a typical agonist of α7nAChR in mammals, was tested to treat fish foodborne enteritis *via* its potential anti-inflammation effect using the zebrafish foodborne enteritis model. After sinomenine’s dietary inclusion, results suggested that there was an alleviation of intestinal inflammation at a pathological level. This outcome was demonstrated through the improved morphology of intestinal villi. At a molecular level, SN suppressed inflammatory cytokines’ expression (especially for *tnf-α*) and upregulated anti-inflammation-related functions (indicated by expression of *il-10*, *il-22*, and *foxp3a*). To systematically understand sinomenine’s intestinal effect on SBMIE, transcriptomic analysis was done on the SBMIE adult fish model. DEGs (sinomenine *vs* soybean meal groups) were enriched in GO terms related to the negative regulation of lymphocyte/leukocyte activation and alpha-beta T cell proliferation, as well as the regulation of lymphocyte migration. The KEGG pathways for glycolysis and insulin signaling indicated metabolic adjustments of α7nAchR mediated anti-inflammatory effect. To demonstrate the immune cells’ response, in the SBMIE larva model, inflammatory gatherings of neutrophils, macrophages, and lymphocytes caused by soybean meal could be relieved significantly with the inclusion of sinomenine. This was consistent within the sinomenine group as CD4^+^ or Foxp3^+^ lymphocytes were found with a higher proportion at the base of mucosal folds, which may suggest the Treg population. Echoing, the sinomenine group’s 16s sequencing result, there were fewer enteritis-related TM7, *Sphingomonas* and *Shigella*, but more *Cetobacterium*, which were related to glucose metabolism. Our findings indicate that sinomenine hydrochloride could be important in the prevention of fish foodborne enteritis at both immune and microbiota levels.

## Introduction

Currently, foodborne intestinal inflammation is a major health and welfare issue in aquaculture. As fishmeal remains an important source of dietary protein in aquaculture diets, its increasing price and decreasing availability have given birth to a replacement of feed made from plant sources of dietary protein, such as soybean meal (1). The side effects resulting from plant-sourced proteins have occurred in many fish species, from carnivorous to herbivorous, such as salmon (2), grouper (3), zebrafish (4, 5), and carp (6). Fish foodborne enteritis also is due to the dysregulation of oral tolerance, which is related to immunoregulation locally at the intestinal mucosa (7). Fish intestinal mucosal surfaces colonized by normal microbiota may elicit immune regulatory functions in gut-associated lymphoid tissues (GALT). Contrastingly, disturbances in these immuno-regulatory functions by an imbalanced microbiota may contribute to the development of disease (8). To prevent enteritis, various feed additives have been designed for modulating the host as well as intestinal bacteria. The effective ingratiates incorporated into the fish diet include probiotics, nutrients, and herbal medicine.

Since the effective components and long-term side-effects of herbal medicine cannot be easily determined, using a natural compound rather than herbal medicine to prevent fish disease may be a better solution for aquaculture (9). A traditional selection of the drugs or additives depends on the experimental result of field tests. Based on the exploration of an omics study, in aquaculture, the pursuit of discovering drugs or additives has developed novel methods, such as the reverse prediction of drugs or additives from the pathway enlightened by nutritionally transcriptomic study. Considering the anti-inflammatory effects of natural compounds, our previous transcriptomic study of grass carp soybean-induced enteritis (SBMIE) has indicated that the intestinal cholinergic anti-inflammatory pathway may work (6) and that the cholinesterase inhibitor galantamine can be used to prevent SBMIE (4). Thus, these findings have indicated the possible existence of the cholinergic anti-inflammatory pathway in the fish gut. Diet inclusion of galantamine could elicit a regulatory function on mucosal inflammation (10).

The preserved intestinal acetylcholine (4) in fish may enhance α7nAChR and had a mediating anti-inflammatory effect on immune cells, such as macrophages, neutrophils, and T cells (11–14). When considering effectors, fish SBMIE showed typical allergic cytokine profiles (15), similar to IBD in humans. For proinflammatory cytokines, TNF-α was highly expressed (especially in the epithelial cells) for accelerating intestinal inflammation (16, 17). The acute inflammation-related TNF-α, IL-1β, and allergic cytokine IL-17A/F have also been reported in fish SBMIE (4, 15, 18). Based on the existence of zebrafish nicotinic acetylcholine receptor α7nAChR (19) as well as α7 nicotinic agonist AR-R17779’s protective effect on colitis (20), α7nAchR signaling would be a promising target to efficiently activate intestinal α7nAChR mediated anti-inflammatory function.

Sinomenine (SN), which is an agonist of α7nAChR (21), could insert into the active site of the α7nAchR structure in mammals (22) and has been identified as an effective component to treat swelling and pain during mucosal inflammation, such as enteritis (23). Sinomenine derivatives have also improved the immunosuppressive activity of its parent natural compound (24). For SN’s effect on intestinal humoral immunity, sinomenine hydrochloride may suppress proinflammatory cytokines (such as TNF-α) and increase anti-inflammatory cytokine IL-10 during DSS-induced enteritis in mice (17). At a cellular level, sinomenine could either promote macrophage reprogramming toward M2-like phenotype (25) or induce the generation of Treg by upregulating the transcriptional factor Foxp3 (22). Recently, the gut microbiome was also found to be a target for regulatory T cell-based immunotherapy (26). In zebrafish, intestinal macrophages have been reported to have the ability to shape gut microbiota (27). These are in line with that SN-altered gut microbiota composition when treating DSS-induced colitis (17).

As an effective model organism, the zebrafish has been used to study enteritis in aquaculture (28, 29). SBMIE is the typical plant-sourced protein-induced inflammation, including in zebrafish, with the typical intestinal pathology (30, 31). To illustrate the immune mechanisms at a cellular level, the immune cell imaging using zebrafish larva provided important clues (28, 32, 33). Using zebrafish to model enteritis (34, 35) has revealed immune mechanisms that were not only the innate immune cells was involved (28, 30) during SBMIE, but also T cells played an important role, especially with a Th17 cytokine profile (15). Zebrafish has become a model for studying gastrointestinal tract-microbe interaction (36). In the zebrafish enteritis model, certain aquatic organisms, as well as natural compounds, have been proved to be effective in orally treating enteritis, such as microalga (28), lactoferrin (33), fucoidan (32). Therefore, zebrafish has become a model for testing ingredients, which could be potentially included in fish feeds.

In this study, sinomenine was tested for its anti-enteritis functions in the zebrafish SBMIE model. Using transcriptomic analysis of hindgut tissue and 16S sequencing of intestinal microbiota, the immune and metabolic processes involved have been revealed. The immune regulatory mechanisms have been assessed by immune cell imaging and regulatory immune cell-related gene expression. Our findings indicate that sinomenine hydrochloride could be used as an effective additive for preventing diet-induced intestinal inflammation and even microbiota dysbiosis in fish.

## Materials and Methods

### Zebrafish

Zebrafish, including both AB wild-type line and fluorescence-labeled lines, including Tg(lyz:DsRED2), Tg(mpeg1:EGFP), Tg(rag2:DsRed), and Tg(lck:lck-eGFP), were purchased from China Zebrafish Resource Center (http://en.zfish.cn/) and were maintained according to standard protocols (37). After crossing neutrophil and macrophage labeled lines, Tg(lyz:DsRED2/mpeg1:EGFP) were obtained, then together with Tg(rag2:DsRed) and Tg(lck:lck-eGFP), three lines of Tg fish were prepared for further experiments. The use of animals in this study was approved by the Animal Research and Ethics Committees of the Institute of Hydrobiology, Chinese Academy of Sciences. All experiments were conducted following the guidelines of the committees.

### Diets and Feeding Trial

The formulation of the experimental diets is shown in **Table 1**. To model SBMIE in adult WT fish (3 mo), diets, as well as the 6-week feeding trial, followed a previously published method (4). For the SBMIE larva model, the feeding trials for both innate and adaptive immune cell imaging followed our recently published protocol (38). In brief, the innate model used 5 dpf larva of Tg(lyz:DsRED2/mpeg1:EGFP) to do a 4-day feeding trial with FM (negative control), SBM (positive control), and drug-included SBM diet (tested group). Afterwards, at 9 dpf, imaging of the hindgut was carried out. The adaptive model used 17 dpf larva of either Tg(rag2:DsRed) or Tg(lck:lck-eGFP) to do a 10-day feeding trial, and then larva at 27 dpf was conducted to imaging analysis. The sinomenine hydrochloride, which is the hydrochloride chemical form of sinomenine (39), was purchased from Xi’an Huilin Biotechnology Co., Ltd in China. Based on our previously researched effective concentration for preventing SBMIE, the dietary inclusion of sinomenine hydrochloride should be in the range of 15–60 ppm (40). Thus, in this study, 35 ppm sinomenine hydrochloride was included in the soybean meal diet to test if it could have any alleviating effect on SBMIE.

**Table 1:** Formulation of experimental diets.

Raw Material g/kg	FM	SBM	SN
Fish meal	555	250	250
Soybean meal	0	500	500
Wheat meal	255	110	110
Starch	50	50	50
Fish oil	30	60	60
Mineral premix^a^	10	10	10
Vitamin premix^b^	10	10	10
Sinomenine	0	0	0.7
Cellulose	90	10	9.3
Gross weight (g)	1,000	1,000	1,000

a Per kilogram of mineral premix (g kg−1): MnSO4·H2O (318 g kg−1 Mn), 1.640g; MgSO4·H2O (150 g kg−1 Mg), 60.530 g; FeSO4·H2O (300 g kg−1 Fe), 23.110 g; ZnSO4·H2O (345 g kg−1 Zn), 0.620 g; CuSO4·5H2O (250 g kg−1 Cu), 0.010 g; KI (38 g kg−1 I), 0.070 g; NaSeO3 (10 g kg−1 Se), 0.005 g. All ingredients were diluted with cornstarch to 1 kg.

b Per kilogram of vitamin premix (g kg−1): retinyl acetate (500,000 IU g−1), 2.40 g; cholecalciferol (500,000 IU g−1), 0.40 g; DL-a-tocopherol acetate (500 g kg−1), 12.55 g; menadione (230 g kg−1), 0.80 g; cyanocobalamin (10 g kg−1), 0.83 g; D-biotin (20 g kg−1), 4.91 g; folic acid (960 g kg−1), 0.40 g; thiamin hydrochloride (980 g kg−1), 0.05 g; ascorhyl acetate (930 g kg−1), 7.16 g; niacin (990 g kg−1), 2.24 g; meso-inositol (990 g kg−1), 19.39 g; calcium-D-pantothenate (980 g kg−1), 2.89 g; riboflavin (800 g kg−1), 0.55 g; pyridoxine (980 g kg−1), 0.59 g. All ingredients were diluted with corn starch to 1 kg.

### Analysis of Sinomenine’s α7nAchR Binding Ability by Molecular Docking

To check if sinomenine could potentially activate zebrafish α7nAchR signaling, PROCHECK server and ProSA web were applied to test the potential binding ability of sinomenine to zebrafish α7nAChR protein, following a previously described method (41, 42).

### Sampling

In adult zebrafish, the hindgut tissue, which is a third of the whole intestine from bend 2 to the anus (43), has been used for sampling. For gene expression analysis, including transcriptome, qPCR, and immunofluorescence (n = 6), the tissues used to isolate RNA for both transcriptomic and qPCR were cut into small pieces and soaked in a 10-fold volume of TRIzol (Invitrogen), and the tissues used to make frozen sections were soaked in a 10-fold volume of 4% PFA (Sigma). For all omics studies, the hindgut from one female and one male adult zebrafish were mixed for each sample (three repetitions) to avoid sex-biased differences for both transcriptome and 16S. The sampling of 16S sequencing analysis followed the method published in previous studies by Deng et al. (44). For the sampling of larvae, only 27 dpf larvae (n = 6) were sampled for HE staining at the meantime of imaging the adaptive immune cells.

### Histological and Immunofluorescence Analyses

To demonstrate the basic pathology of intestinal mucosa, both hematoxylin eosin (HE) staining and immunofluorescence were performed using hindgut tissue slices of 10 μm. Then, the zebrafish anti-CD4-1 antibody (GeneTex), which can immune-stain the T helper cell, was applied during immunofluorescence analysis to further illustrate the inflammatory aggregation or infiltration of lymphocytes in different intestinal layers or villi locations in the hindgut (4). The zebrafish anti-foxp3 antibody (GeneTex) was also used to identify the regulatory lymphocytes in intestinal mucosal folds (45). Meanwhile, DAPI was used parallelly to stain the nucleus.

### Library Preparation and Sequencing for Transcriptomic Data

For the hindgut’s RNA (n = 6), transcriptomic analysis was completed to reveal the gene expression profile systematically in adult fish from all groups, including the negative control (FM group), the positive control (SBM group), and the tested drug inclusion group (SN group). The procedure of the gene library preparation and sequencing for transcriptome followed previously published methods (46). Briefly, sequencing libraries were generated using NEBNext R UltraTM RNA Library Prep Kit for Illumina R (NEB, USA) following the manufacturer’s recommendations, and the library quality was assessed on the Agilent Bioanalyzer 2100 system. The library preparations were sequenced on an Illumina platform (NovaSeq 6000), and 150 bp paired-end reads were generated.

### Transcriptome Assembly, DEG Analysis, and Functional Annotation

After the clean data were mapped onto the reference genome by HISAT2 (version 2.1.0) (47), the transcript was assembled using StringTie (Version V1.3.1c) (48). Salmon (version 0.12.0) (49) was used to calculate the gene expression. | log2FoldChange | > 1 and padj < 0.05 is considered to be differentially expressed genes (DEG), using DESeq2 1.24.0 (version) to analyze differential expression (50). For annotations, enrichment analysis of both GO terms and KEGG pathways was performed using clusterProfiler (version 3.12.0) (51), and *p* < 0.05 was considered to be a significant enrichment. In the case of the parameters used not being listed, default parameters were used.

### RNA Extraction and qPCR Analysis

Principally, the RNA extraction and qPCR analysis of the zebrafish intestinal tissues were done according to previously published procedures (4, 6). In brief, TRIzol (Invitrogen) was used to extract RNA, and then the isolated RNA was reverse-transcribed into cDNA using HiScript III 1^st^ Strand cDNA Synthesis Kit (Vazyme). In addition, all the RNA extraction was done immediately after sampling, or at the same time for sampling to do an omics study. Genes of interest, including both randomly selected DEGs and genes involved in α7nAChR, mediated anti-inflammatory function were validated for their expressional changes by qPCR. Specifically, genes of both intestinal pro-inflammatory factors (transcription factor *nf-κb*, cytokines *il-1β*, *tnf-α*, *il17a/f3*, and CD4) and anti-inflammatory factors (cytokines *tgf-β1a*, *il-10*, *il22* and transcription factor *foxp3a* and *foxp3b*) have been checked for SN-induced α7nAChR-mediated anti-inflammatory effect in intestine. Meanwhile, *rpl13a* was used as the internal reference. The primers used to amplify these genes are listed in **Table 2**.

**Table 2:** qPCR primers used in enteritis-related cytokine gene expression analysis.

Gene Name	Forward sequence	Reverse sequence
il-1β	5’-TGGACTTCGCAGCACAAAATG-3’	5’-GTTCACTTCACGCTCTTGGATG-3’
nf-κb	5’-GATCATCGAGCAGCCTAAATC-3’	5’-CCCACTGTAGTTGTGAACCCT-3’
tnf-α	5’-GCGCTTTTCTGAATCCTACG-3’	5’-TGCCCAGTCTGTCTCCTTCT-3’
il-17a/f3	5’-AAGATGTTCTGGTGTGAAGAAGTG-3’	5’-ACCCAAGCTGTCTTTCTTTGAC-3’
tgf-β1a	5’-TGTACCCGCAATCCTTGACC-3’	5’-CCGACTGAGAAATCGAGCCA-3’
il-10	5’-CACTGAACGAAAGTTTGCCTTAAC-3’	5’-TGGAAATGCATCTGGCTTTG-3’
il22	5’-GATGACTGATACAGCACGAAA-3’	5’-CATTGATGCAGCAGGAACCT-3’
foxp3a	5’-GCCTCCATGATACGATGGGCAAT-3’	5’-CCTTCCTTCAACACGCACAA-3’
rpl13a	5’-TCTGGAGGACTGTAAGAGGTATGC-3’	5’-AGACGCACAATCTTGAGAGCAG-3’

### *In Vivo* Imaging of Immune Cells in Zebrafish Larvae

*In vivo* imaging of immune cells was completed following previous studies of zebrafish larvae imaging (15, 28, 32), with some modified conditions, such as the incubating solution, exact time, and microscopy. Imaging of innate immune cells, including neutrophils (lyz labeled) and macrophages (mpeg labeled) in the hindgut (tail part) of Tg(lyz:DsRED2/mpeg1:EGFP) larvae (n = 10), was through referencing previously published methods (38). Larvae were fed with experimental diets (diets for FM, SBM, SB groups, which were crushed and passed through an 80-mesh sieve) between 5 to 8 dpf. At 9 dpf, the Tg(lyz:DsRED2/mpeg1:EGFP) larvae were anesthetized by MS222 and then embedded in 1% LMP Agarose (Invitrogen), dissolved in Danieau’s solution (52) to conduct imaging analysis. For imaging adaptive immune cells, including immature (rag2 labeled) and mature lymphocytes (lck labeled) in the hindgut (tail part) of Tg(rag2:DsRed) and Tg(lck:lck-eGFP) larvae (N = 10), we followed previously published methods (38). After feeding 12 days of Larval AP100 Diet (Zeigler) from 5 to 16 dpf, as well as 10 days of experimental diets from 17 dpf, at 27 dpf the larvae were anesthetized and embedded in 1% LMP Agarose (Invitrogen), dissolved in Danieau’s solution (52). The Tg(rag2:DsRed) or Tg(lck:lck-eGFP) larvae were observed under a Leica SP8 microscope. For the intestinal imaging of larvae, a composition of several stacks was merged to generate the picture for the whole tail part of the intestine.

### 16S rRNA Gene Sequencing of Intestinal Microbiota

The composition of bacterioplankton was analyzed using sequencing of 16S rRNA gene amplicons. DNAs from the six repetitions were extracted for each treated sample. Then the V3V4 regions of the 16S rRNA genes, which can yield accurate taxonomic information, were amplified with the primer set 338F (5’- ACTCCTACGGGAGGCAGCA-3’)/806R (5’-GGACTACHVGGGTWTCTAAT-3’). After the libraries were built, the paired-end 250-nucleotide reads were yielded using the Illumina NovaSeq platform, according to the manufacturer’s instructions. Raw sequences were then trimmed, quality filtered, denoised, merged, chimera and dereplicated using the DADA2 plugin (53). Reads with 100% nucleotide sequence identity across all samples were assigned to operational taxonomic units (OTUs), and taxonomy was assigned to Non-singleton amplicon sequence variants (ASVs) using the classify-sklearn naïve Bayes taxonomy classifier in the feature-classifier plugin (54) against the Greengenes reference database (version 13.8, http://greengenes.secondgenome.com/) (55).

### Statistical Analysis

The HE-stained intestinal villi, immunofluorescence signals, as well as imaging results of immune cells in larvae were quantified by Image J software. T-tests were used to assess differences for most parameters for staining and imaging results, with a false discovery rate adjusted *p* < 0.05 or 0.01. For the quantification of immunofluorescence signals, the quantitative analysis method followed a previously published study (6, 56) with the modification to calculate the positive cell NO. per villi, which equaled to that the account of number was divided by the average length of intestinal villi. Then, the data were processed using Excel and SPSS 22.0. After eliminating abnormal data by Grubbs’ test, one-way ANOVA was used to analyze the variance. The minimum significant difference method was used for multiple comparisons. Means ± Standard Deviation was used to generate bar. *p* < 0.05 was judged as significant difference, meanwhile *p* < 0.01 as extremely significant difference. The rearranged data from the pathological or immune analyses were applied to GraphPad Prism version 5.0 software for all graphs. For the correlation analysis to compare fold changes between results of qPCR and DEGs, data were processed in EXCEL. The plots and diagrams were displayed by ggplot2 (2.2.1) using R language.

## Results

### The Validation of α7nAchR Binding Ability of Sinomenine

The results of molecular docking showed that sinomenine can bind to the same binding pocket of zebrafish α7nAChR as the positive control Epibatidine, a typical agonist of α7nAChR (**Figure S1**). In this pocket, sinomenine could form hydrogen bonds with several amino acids, including tryptophan, phenylalanine, and lysine. The binding energy of sinomenine to zebrafish α7nAChR was −29.62 kJ/mol, which was comparable to that of positive control Epibatidine (−37.15 kJ/mol).

### Effect of Sinomenine on Intestinal Pathology in SBMIE Adult Model

**Figure 1:**
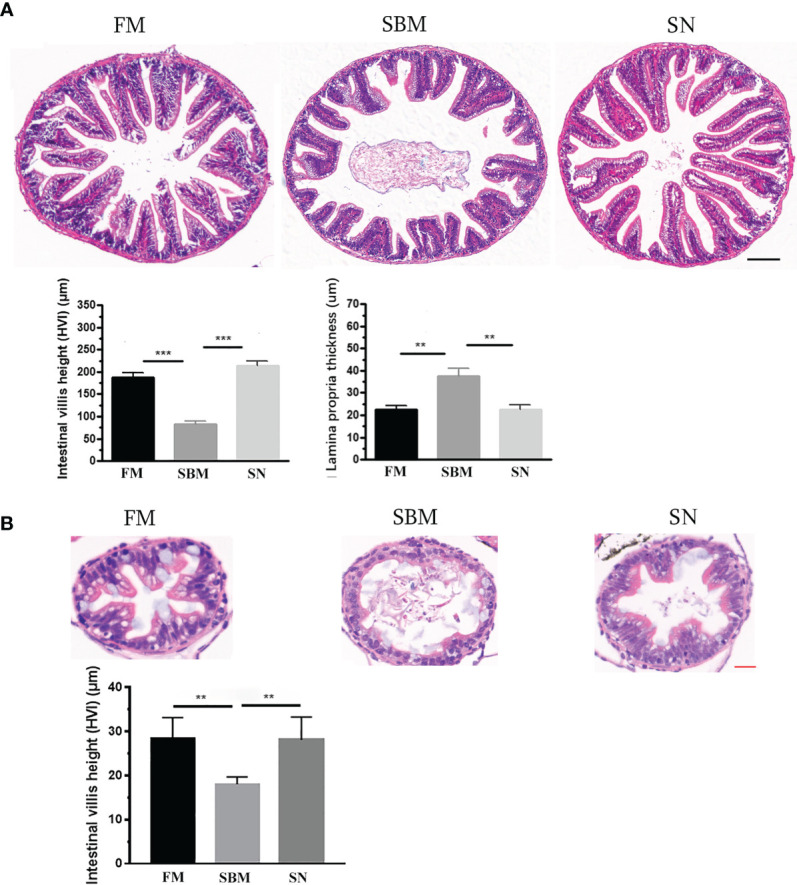
Pathological analysis of sinomenine’s effect on the intestinal mucosal fold. **(A)** HE staining of intestinal mucosa in adult fish fed with FM, SBM, and SN diets. Scale bar: 100 μm. **(B)** HE staining of intestinal mucosa in 27 dpf larva fed with FM, SBM, and SN diets. FM, fish meal diet; SBM, soybean meal diet; SN, sinomenine supplementary SBM diet. Scale bar: 20 μm. ** represented *p* < 0.01, and *** represented *p* < 0.001.

The inclusion of sinomenine at 35 ppm significantly prevented pathological changes in the hindgut. The HE staining result demonstrated the relief of intestinal lesion at mucosal fold level upon sinomenine dietary inclusion, compared with the SBM group. Quantitative analyses of the histological parameters in the SBMIE adult model reflect that in the SN group the height of intestinal villi increased while the thickness of lamina propria (LP) decreased very significantly (*p* < 0.01) (**Figure 1A**). The increased height of intestinal villi (*p* < 0.01) in the SN group was revealed in the hindgut at 27 dpf in the larva model (**Figure 1B**), compared to the very shortened mucosal fold in the SB group. Specifically, in the LP layer of intestinal villi, the widened top with lymphocyte infiltration indicated by the blue hematoxylin-stained nuclei in SN group was reduced compared to that in the SBM group.

### Lymphocyte-Related Intestinal Protein Expression

**Figure 2:**
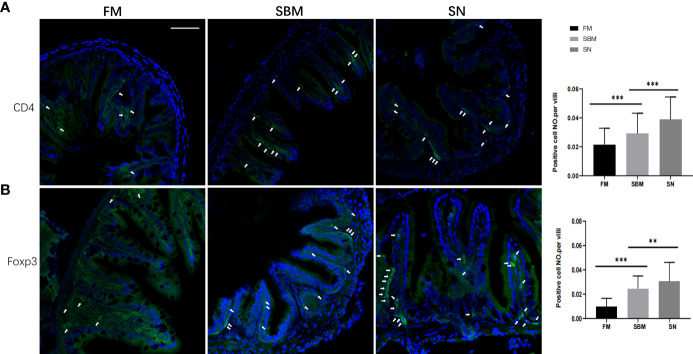
Lymphocyte-related intestinal protein expression reflected by immunofluorescent signals. CD4^+^ **(A)** or Foxp3^+^ **(B)** immunofluorescence signals (green) in adult fish fed with FM, SBM, and SN diets. The quantification of the ratio, which was calculated by dividing positive cell number with intestinal villi’s length, was shown beside the typical images (at the right side). Typical signals are indicated by arrows. The blue signals represented DAPI-stained cell nucleus. FM, fish meal diet; SBM, soybean meal diet; SN, sinomenine supplementary SBM diet. Scale bar: 100 μm. ** represented *p* < 0.01, and *** represented *p* < 0.001.

To reveal the mechanisms of the lymphomonocytes involved, T helper cells, which are at the nexus of the innate and adaptive arms of the immune system (57), have been checked for related protein expressions using immunofluorescent analysis. As the surface marker of T helper lymphocytes and expressed protein on certain populations of phagocytes in zebrafish (57), the immunofluorescence results of intestinal CD4-1 signals showed that the inflammatory aggregation in the widened LP layer of the hindgut, especially at the top of villi in the SBM group. While in the SN group, though that the positive cell NO. per villi of CD4 signals increased, there are fewer signals in the top of villi, but a large proportion of CD4^+^ cells were found at the base of the mucosal fold (**Figure 2A**).

Further, to reveal the Treg’s involvement, an immune-stained transcriptional factor reflected that there were brighter and more Foxp3^+^ cells in the SN group, and a substantial proportion of Foxp3^+^ cells was found at the base of the villi in the SN group, compared to SBM group (**Figure 2B**). In the SBM group, Foxp3^+^ cells were also found at the edge of basal plasmacytosis (**Figure 2B**). While it was interesting to notice many vasally located Foxp3^+^ cells in the SN group (**Figure S2**). Considering the recovered length of intestinal villi, the positive cell NO. per villi of Foxp3 in the SN group was lower compared to the SBM group, yet still higher than the FM group (**Figure 2B**).

### qPCR Analysis of DEGs and Intestinal Pro- or Anti-Inflammatory Factors

**Figure 3:**
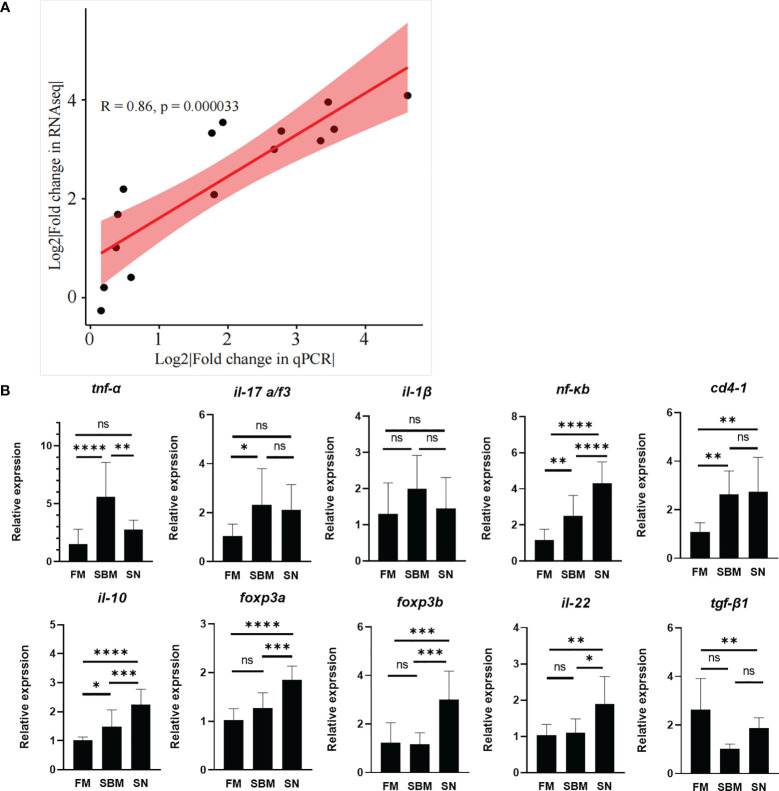
qPCR validation. **(A)** qPCR validation of transcriptomic data; **(B)** qPCR analysis of enteritis-related pro-/anti-inflammatory factors, which could reflect α7nAchR-mediated anti-inflammatory effect in fish. "ns" represented p > 0.05, * represented *p* < 0.05, ** represented *p* < 0.01, and *** represented *p* < 0.001.

The raw data of current transcriptomic analysis have been submitted to the Genome Sequence Archive (GSA) database (http://gsa.big.ac.cn/index.jsp) with the BioProject identifier <PRJCA005917> and data ID <CRA004582>. The close correlation (R > 0.08) of fold changes between fold changes of qPCR result and DEGs (**Figure 3A** and **Table S1**) proved the successful decoding of gene expression by the current transcriptomic analysis. Specifically, the immunoregulatory role of SN was reflected by the expression of pro-/anti-inflammatory factors (**Figure 3B**). The pro-inflammatory cytokine genes’ result (**Figure 3B**, upper) showed that in the SN group *tnf-α* was very significantly downregulated compared to the SBM group, and even dropped to a similar level to the FM group. *il-1β* and *tnf-α* were downregulated with non-significant trends, yet, *nf-κb*, *il17a/f3*, and *cd4* were almost not changed compared to the SBM group. The anti-inflammatory factor genes’ result (**Figure 3B**, lower) showed that except for the not significantly upregulated *tgf-β1a*, *il-10*, *il22*, *foxp3a*, and *foxp3b* were significantly upregulated.

### Enriched GO Terms and KEGG Pathways of Intestinal DEGs

**Figure 4:**
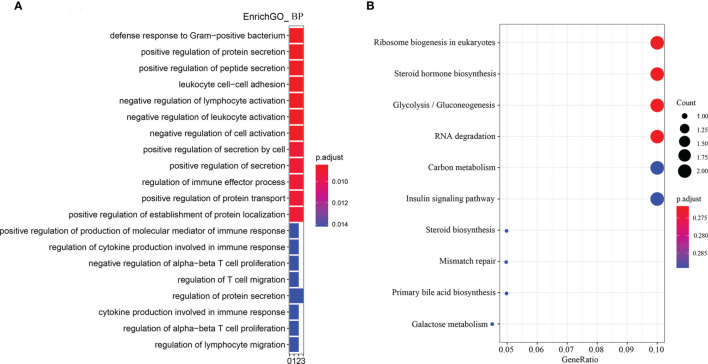
Enriched GO terms and KEGG pathways for DEGs from the comparison between the SN and SBM groups in the zebrafish SBMIE adult model. **(A)** The GO terms of biological process; **(B)** the KEGG pathways. SBM, soybean meal diet; SN, sinomenine supplementary SBM diet.

According to the enriched biological process GO terms of DEGs from a comparison between SN *vs* SBM groups (with details in **Table S2**), there were many immune cells or process-related terms (**Figure 4A** and **Table S3**). For immune cells, there were “leukocyte cell-cell adhesion,” “negative regulation of lymphocyte activation,” “negative regulation of leukocyte activation,” “negative regulation of alpha-beta T cell proliferation,” “regulation of T cell migration,” “regulation of alpha-beta T cell proliferation,” “regulation of lymphocyte migration.” For the immune process, there were “regulation of immune effector process,” “positive regulation of production of molecular mediator of immune response,” “regulation of cytokine production involved in immune response,” and “cytokine production involved in immune response.” Meanwhile, among revealed KEGG pathways (**Figure 4B**, **Table S4**), a high gene ratio existed in six pathways, which contained metabolic-related “glycolysis/gluconeogenesis” and “insulin signaling pathways.”

### Effect of Sinomenine on Intestinal Immune Cell in Zebrafish Larvae

**Figure 5:**
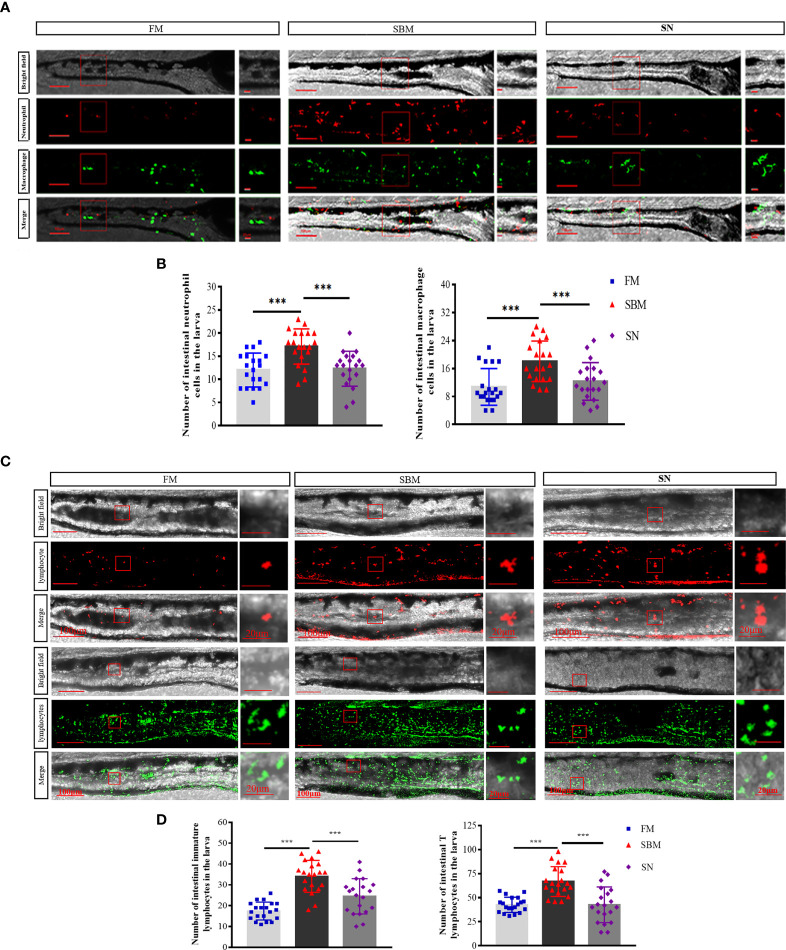
Imaging analysis of immune cells in the hindgut of the zebrafish SBMIE larva model. **(A)** Hindgut innate immune cells, including neutrophils (lyz-DsRed labeled cells) and macrophages (mpeg-EGFP labeled cells); **(B)** the histogram of lyz or mpeg signals in the hindgut of larva. **(C)** Hindgut adaptive immune cells, including immature lymphocytes (rag2-DsRed-labeled red cells) and mature T lymphocytes (lck-EGFP-labeled green cells). **(D)** The histogram of rag2^+^ or lck ^+^ signals in the hindgut of larva. FM, fish meal diet; SBM, soybean meal diet; SN, sinomenine (35 ppm) supplementary SBM diet. The significant differences were indicated by *p* value, and *** represented *p* < 0.001. The scale bar in whole hindgut pictures was 100 μm, while the scale bar in the enlarged view was 20 μm.

At a cellular level, for innate immune cells, sinomenine could significantly (*p* < 0.01) relieve inflammatory aggregation for both neutrophils (lyz^+^ cells) and macrophages (mpeg^+^ cells) (**Figures 5A, B**) in the hindgut of the larva. For adaptive immune cells, sinomenine could significantly (*p* < 0.01) inhibit the inflammatory aggregation and immune synapse formation of both rag2^+^ immature lymphocytes and lck^+^ mature T lymphocytes (**Figures 5C, D**). Specifically, the formation of immune synapses of mpeg^+^ and rag2^+^ cells in the SBM group could be inhibited in the SN group.

### Effect of Sinomenine on Microbiota OTU and Taxa Composition in Hindgut

**Figure 6:**
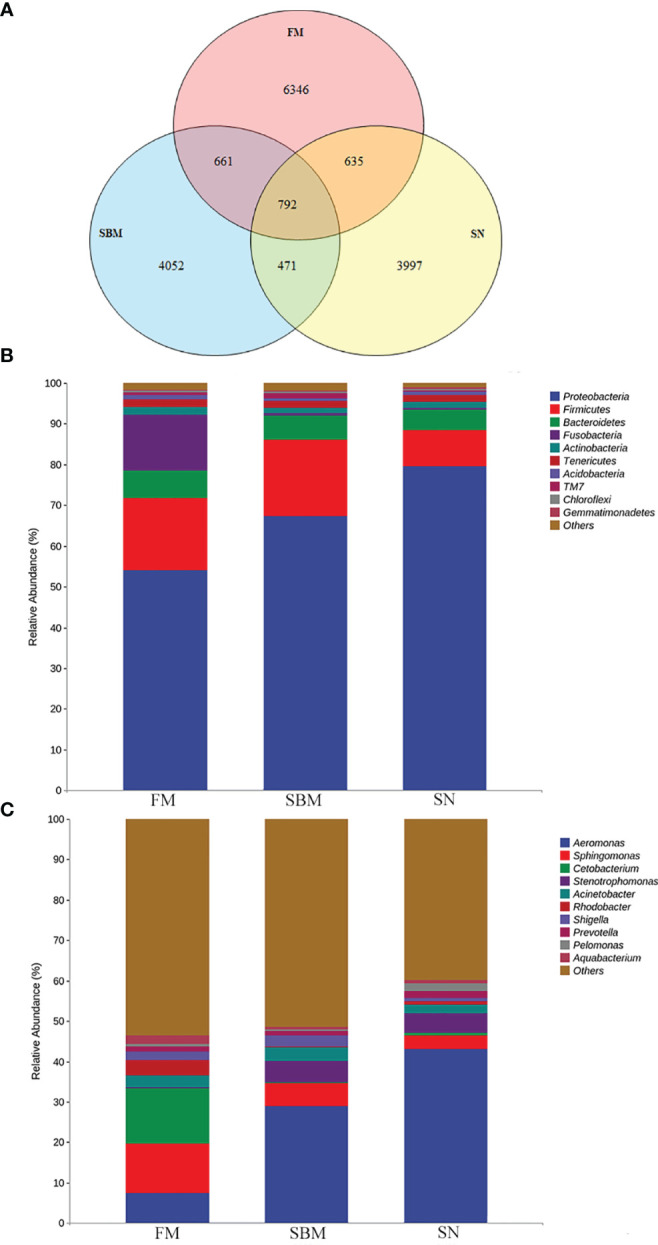
Analysis of intestinal microbiota OTU and taxa composition influenced by sinomenine inclusion in zebrafish SBMIE adult model. **(A)** Venn diagram of OTU in the FM, SBM, and SN groups; **(B)** hindgut bacteria composition at phylum level; **(C)** hindgut bacteria composition at the genus level. FM, fish meal diet; SBM, soybean meal diet; SN, sinomenine supplementary SBM diet.

The identified OTU in the hindgut of adult zebrafish for FM, SBM, and SN groups were 8,434, 5,976, and 5,895, respectively. As indicated by the Venn diagram (**Figure 6A**), the overlapping between the sinomenine and FM groups covered 635 OTUs, while the overlapping between the sinomenine and SBM groups contained 471 OTUs. To estimate the abundance of taxa, currently revealed OTUs could be identified by a lot of phyla. Among the most abundant 10 phyla, compared to the SBM groups, in the SN group, the increased phyla included Proteobacteria, Actinabactieria, Acidobacteria, and Gemmatimonadetes, while decreased phyla included Firmicutes, Bacteroidetes, Fusobacteria, and TM7 (**Figure 6B**). Moreover, at a genus level (**Figure 6C**), among the 10 genera of most abundance, compared to SBM groups, in the SN group increased genera included *Aeromonas*, *Cetobacterium*, *Rhodobacter*, *Prevotella*, *Pelomonas*, and *Aquabacterium*, while decreased genera included *Sphingomonas*, *Acinetobacter*, and *Shigella*. The raw data of the current 16S sequencing analysis have been submitted to the Genome Sequence Archive (GSA) database (http://gsa.big.ac.cn/index.jsp) with the BioProject identifier <PRJCA005917> and data ID <CRA004611>.

## Discussion

In the present study, efforts were made to activate the intestinal local immune modulation by α7nAChR-mediated anti-inflammatory function to help treat fish foodborne enteritis. The results indicated sinomenine, which could closely bind to the zebrafish α7nAChR, reduced fish foodborne enteritis pathology at both the anti-inflammatory and metabolic levels paralleled by ameliorating dysbiosis of intestinal microbiota.

Enlightened by the structure-guided medicine discovery approach (58), the sinomenine’s protective role against SBMIE was revealed in the fish foodborne enteritis model. Pathologically, the current data showed that sinomenine did work in the gut. Based on the successful modeling of SBMIE in adult or larval zebrafish as in our previously published papers (19, 20), the typical intestinal pathology, such as villus atrophy and basal cell hyperplasia, has also been observed in the positive control (SBM group) and was found to be relieved by the present results in the SN group. Although in mice, intestinal neutrophil recruitment could be inhibited *via* M2AChR (59), the currently used α7nAchR-specific agonist SN also suppressed fish neutrophil aggregation in the hindgut. This may be the downstream effect of suppressed TNF-α by SN. For the macrophage and T cell signals reflected by imaging analysis as well as immune-stained CD4-1 signals, this may result from SN-evoked α7nAChR-mediated anti-inflammatory macrophage and Treg’s effects, as well as non-immune function, such as promoting local tissue repair (60) and preserving the integrity of the epithelial barrier (61). The tissue repair was also suggested by the upregulated trend of TGF-β. The current study revealed a large proportion of Foxp3a^+^ cells are located in the basal part of LP and even in the intestinal vasal. This was in line with the HE results that in the SN group the lymphocytes infiltration mainly at the villi top was reduced compared to the SBM group.

At a molecular level, the revealed DEGs in transcriptomic analysis already provided signs for the involved mechanisms in SN’s anti-inflammation effect. As for the upregulated genes, the peptidoglycan recognition protein may play a role similar to the pore-forming C-type lectin; thus, its higher expression was associated with enhanced intestinal mucosal immunity (36). The higher level of *intelectin 3*, which was reported to be involved in the protective mycobacterial immune response, may be a beneficial mucosa factor against dysbiosis. *Enolase*, a neuronal marker (62), may indicate enhanced participation of enteritic neurons upon sinomenine’s stimulation. Significant upregulation of both *foxp3a* and *foxp3b*, which was responded to specifically in the fish intestine (63), may indicate both the impute and local differentiation of Treg in the hindgut. On the other hand, among the downregulated genes, there was a lower expression of TRIM9, which may suggest lower rates of macrophage migration (64). Downregulated Thy-1 in the SN group potentially suggested fewer gut intramucosal lymphocytes (65).

The currently revealed SN’s immune effect was reflected at both innate and adaptive levels. Imaging of neutrophils and macrophages echoed with leukocyte-related GO terms; meanwhile, imaging of lymphocytes was coincident with lymphocyte-related terms. As to the enteritis-related cytokine, the significantly suppressed *tnf-α*, which is at the upstream of intestinal pro-inflammatory cytokines (66) (such as the currently revealed downregulated *il-1β* though not significantly), may play an important role in SN’s ameliorating effect. Compared to the SBM group, the decreased number of both neutrophils and macrophages may result from the significantly restrained *tnf-α* expression. For macrophages, fewer immune synapse-like structures in the SN group may suggest the inhibition of innate immune cells’ migration and phagocytic activity (67, 68), and the inhibition of the M1 phase at the mucosa (60). These results of fish macrophages were in line with the findings in mammals that the interaction with α7nAChR expressed on macrophages leads to a reduction of pro-inflammatory cytokines (13). In addition, for intestinal mucosa immunity, the significantly upregulated *il-22* (qPCR result) together with upregulated *muc2* in all DEGs (highlighted in **Table S2**) may indicate the protective function to maintain host epithelium integrity (69, 70).

From an adaptive view, hindgut lymphocytes were indicated as the SN’s effective target, based on many lymphocytes (especially T cell) related biological process GO terms and imaging result of lymphocytes in comparison between SN and SBM groups. In general, hindgut lymphocytes’ responses may be limited by SN, as that the number of lymphocytes was reduced and immune synapse like structure in Rag^+^ cells in 27 dpf larvae’s hindgut disappeared in the SN group. Yet, the increased intestinal CD4 at a protein level, but not RNA level, maybe due to the vesicular input caused by SN-induced systemic immune’s response (71). The basal location of CD4 together with upregulated immune-regulated factors (*il-10*, *foxp3a*, and *foxp3b*) may be consistent with the location in the basal part of LP or mucosal fold was a feature of only Treg among Th populations (72). SN stimulated upregulated both TGF-β and Foxp3 echoed with that grass carp TGF-β1 could upregulate Foxp3 expression (73). Regarding the glucose metabolism and insulin-related KEGG pathways, as both the lower glucose values and insulin therapy seemed to be anti-inflammatory (74), these pathways could be considered as the metabolic effects of intestinal α7nAChR-mediated anti-inflammatory function in fish.

Coincident with recent findings that SN’s effect could be limited by antibiotic’s disturbing the intestinal microbiota (75), the current study used the immunomodulator SN in the fish diet, which caused an altered OTU and taxa composition in the hindgut. This actinobacterium could produce active metabolites against pathogenic microorganisms (76), increasing Actinabacteria and, as a result, indicating a protective factor in the SN group. Increased Acidobacteria in the SN group may indicate better protein utilization since that Acidobacteria was revealed as a major component of intestinal microbiota in carnivorous fish (77). The decreased Bacteroidetes, which was responsible for carbohydrate degrading in the intestine (78), may indicate less importance for starch utilization in the SN group. The decreased TM7 suggested less intestinal inflammation and that intestinal TM7 bacterial phylogenies may be a promoter of inflammation for IBD (enteritis in humans) (79). This finding was echoed with the result at a genus level, as that decreased *Sphingomonas*, which has been revealed as a disease biomarker in zebrafish (80), as well as *Shigella*, which has been found associated with CD (enteritis in humans) (81). Both of these results have indicated relief of enteritis. Upregulated probiotic *Rhodobacter* (82, 83) may be a protective factor. The increased *Cetobacterium*, which suggested improvement of fish carbohydrate utilization (84), was echoed with the KEGG pathway “glycolysis/gluconeogenesis” in the SN group.

**Figure 7:**
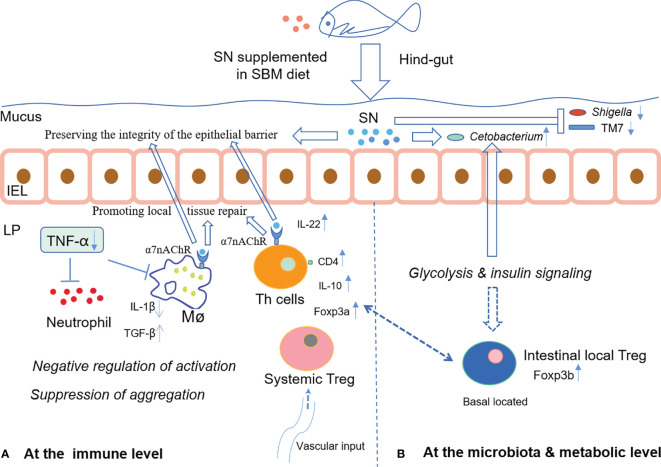
The hypothetical mechanism of SN’s α7nAchR-mediated anti-inflammatory effect on the intestinal immune barrier during foodborne enteritis in the fish hindgut **(A)** at the immune level; **(B)** at the microbiota and metabolic level. IEL, intestinal epithelial layer; LP, lamina propria layer; Mø, macrophage; Th, T helper; Treg, regulatory T cell.

In summary, dietary supplementation of sinomenine hydrochloride could enhance intestinal immune barrier function *via* both inhibiting aggregation of immune cells and changing immune cells’ status possibly through glucose metabolism, whilst ameliorating microbiota dysbiosis to prevent foodborne enteritis in fish. The hypothetical mechanism of SN’s α7nAchR-mediated anti-inflammatory effect on the intestinal immune barrier during foodborne enteritis in fish hindgut is shown in **Figure 7**. Since that in fish, the effective concentration for diet inclusion is very low and sinomenine hydrochloride is cheap, the commercial use of sinomenine hydrochloride could be expected to treat foodborne enteritis in cultured fish.

## Data Availability Statement

The data presented in the study are deposited in the Genome Sequence Archive (GSA) database (http://gsa.big.ac.cn/index.jsp) with the BioProject identifier PRJCA005917 and data ID CRA004582 and CRA004611.

## Ethics Statement

The animal study was reviewed and approved by the Animal Research and Ethics Committees of the Institute of Hydrobiology, Chinese Academy of Sciences. Written informed consent was obtained from the owners for the participation of their animals in this study.

## Author Contributions

NW conceived the project. JX, NW, and XZ wrote the manuscript. ML, JX, JS, XZ, YL, and YC performed the experiments. JX, ML, WY, YD, NW, WZ, and YL did data analysis. BU did language proofreading. The manuscript was read and approved by X-QX. All authors contributed to the article and approved the submitted version.

## Funding

This work was funded by grants from National Natural Science Foundation of China (31872592).

## Conflict of Interest

The authors declare that the research was conducted in the absence of any commercial or financial relationships that could be construed as a potential conflict of interest.

## Publisher’s Note

All claims expressed in this article are solely those of the authors and do not necessarily represent those of their affiliated organizations, or those of the publisher, the editors and the reviewers. Any product that may be evaluated in this article, or claim that may be made by its manufacturer, is not guaranteed or endorsed by the publisher.
