# Supplementary material for: Sinomenine Hydrochloride Ameliorates Fish Foodborne Enteritis via α7nAchR-Mediated Anti-Inflammatory Effect Whilst Altering Microbiota Composition
Source: Front Immunol. 2021 Nov 23;12:766845. doi: 10.3389/fimmu.2021.766845 (PMC8650311; doi:10.3389/fimmu.2021.766845)
Supplement: Supplementary file 1 [file DataSheet_1.pdf]

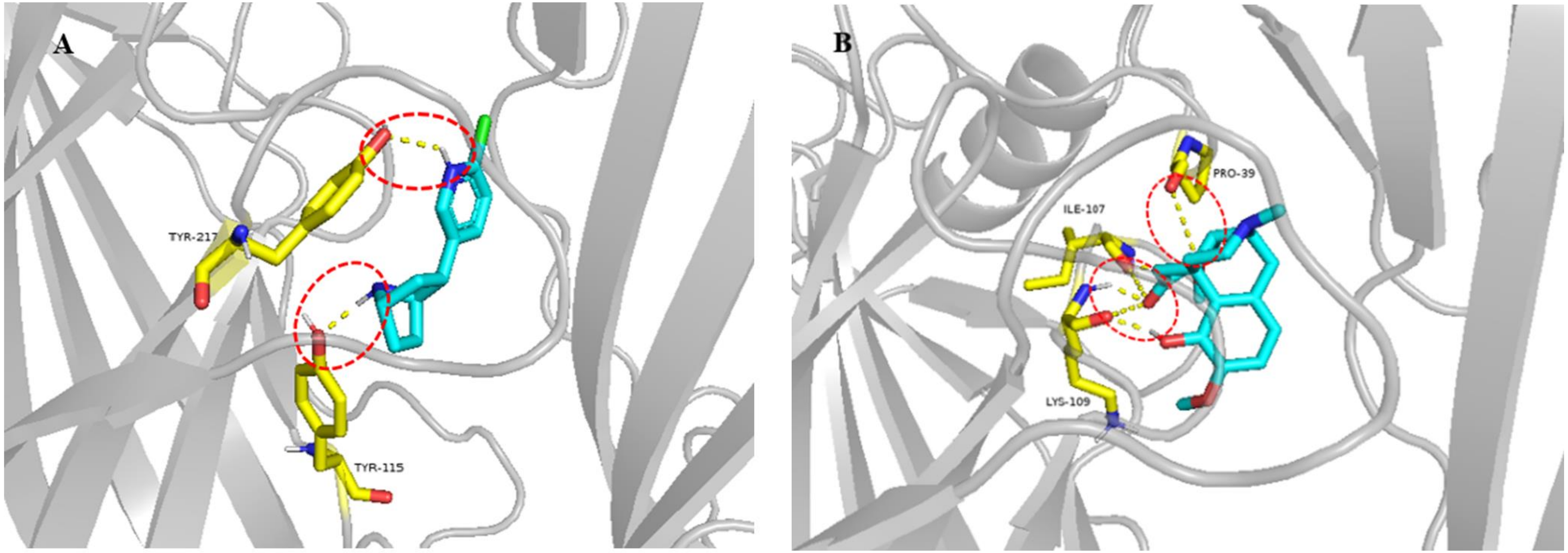

Figure S1 Molecular docking of sinomenine to zebrafish α7-nAChR protein. (A) the docking of epibatidine (positive control) to α7-nAChR's activation pocket; (B) the docking of sinomenine to α7-nAChR's activation pocket. The red circle represents the interaction between the molecule and amino acid residue through hydrogen bonds.

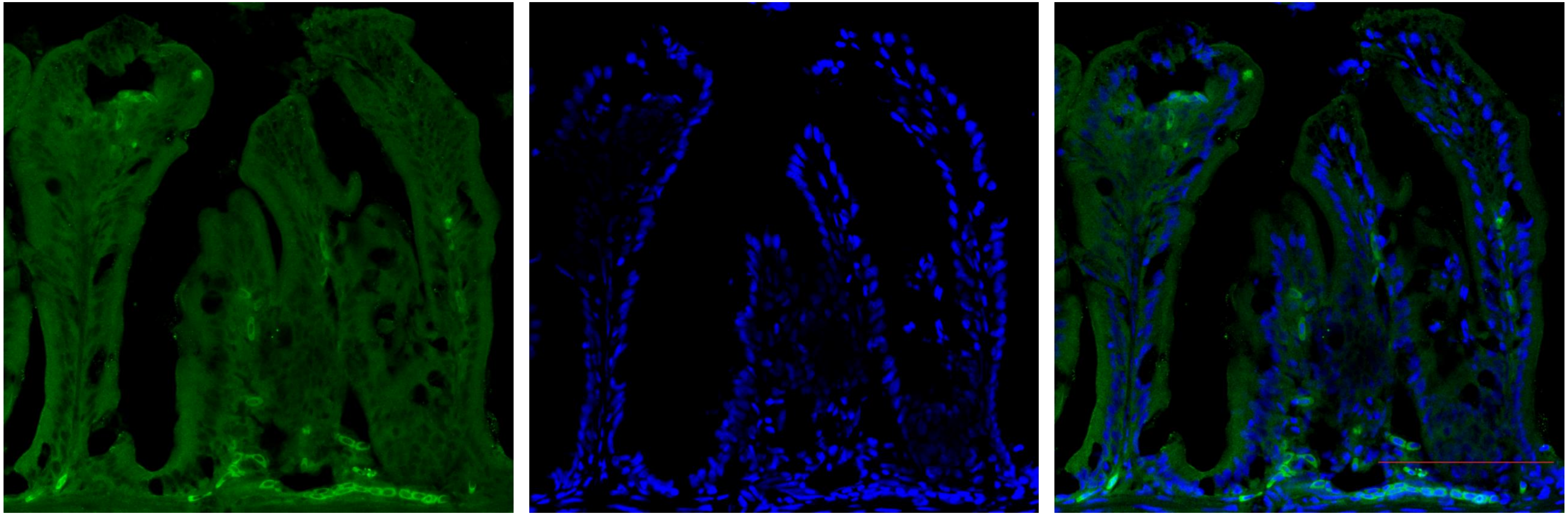

Figure S2 Foxp3 positive intestinal signals in SN group. The green signals represent Foxp3+ cells and the blue signals represent DAPI-stained nuclei. Scale bar: 100 μm.
